# Two Draft Genome Sequences of *Sphingobacterium* sp. Strains Isolated from Honey

**DOI:** 10.1128/genomeA.01364-17

**Published:** 2017-11-30

**Authors:** Alexandra Veress, Tímea Wilk, János Kiss, Péter P. Papp, Ferenc Olasz

**Affiliations:** Agricultural Biotechnology Institute of the National Agricultural Research and Innovation Centre, Gödöllő, Hungary

## Abstract

Here, we report two annotated draft genome sequences of *Sphingobacterium* sp. strains isolated from honey. The genomes of strains 1.A.4 and 1.A.5 show a limited similarity to each other and to genomes of other *Sphingobacterium* species, indicating that these isolates may represent new species.

## GENOME ANNOUNCEMENT

*Sphingobacterium* species are Gram-negative, nonmotile, and nonfermentative rods (sometimes cocci) belonging to the family *Sphingobacteriaceae*. Sphingobacteria have been isolated from diverse niches. They commonly occur in soil, plants, food, and animal guts (1–3) and have been reported to cause infections and sepsis in humans (4). Sixty *Sphingobacterium* sp. whole-genome sequences are available in databases, and four of them are complete sequences. The two *Sphingobacterium* sp. strains presented here, 1.A.4 and 1.A.5, were isolated from the same honey sample obtained in 2014 from Transylvania, Romania, representing the first *Sphingobacterium* isolates from a sugar-rich environment.

The previous 16S rRNA gene sequence analysis suggested that strains 1.A.4 and 1.A.5 can be classified as belonging to the genus *Sphingobacterium*, which was confirmed by the analysis of six further genes (*dnaJ*, *groEL*, *gyrA*, *gyrB*, *recA*, and *rnaP*). In order to investigate their whole genomes, total DNA was isolated from the two strains, and 600- to 630-bp fragment libraries were prepared by UD GenoMED (Debrecen, Hungary). The 2 × 300-bp Illumina paired-end sequencing was performed on an Illumina MiSeq platform by the Department of Biochemistry and Molecular Biology, University of Szeged, Hungary, as a custom service. The read numbers were 1.5 million and 930,000, and the estimated coverages of the whole genomes were 112× and 61× for strains 1.A.4 and 1.A.5, respectively. The reads were *de novo* assembled using the A5-miseq (5) platform. The total lengths of the contigs for strains 1.A.4 and 1.A.5 were 4,005,031 and 4,547,892 bp, and their GC contents were 43.54% and 40.49%, respectively. Annotation of the assembled genome sequences was performed using the Rapid Annotations using Subsystems Technology (RAST) server (6), setting the genetic code to 11 (*Archaea*, bacteria). In the whole genomes of strains 1.A.4 and 1.A.5, 3,673 and 4,241 annotated genes, 112 and 110 tRNAs, and 27 and 22 rRNAs were identified, respectively.

Pairwise comparison (7) of the sequences revealed only 78.55% similarity between strains 1.A.4 and 1.A.5, while their genome sequences showed 81.34% and 84.11% similarities, respectively, to that of *Sphingobacterium spiritivorum* ATCC 33300, which appeared to be the closest neighbor based on the RAST analysis. Likewise, low similarities were also observed when the genomes of strains 1.A.4 and 1.A.5 were compared with those of other *Sphingobacterium* genomes (8), suggesting the flexibility of these bacteria. The relatively low similarity of the genome organization of strains 1.A.4 and 1.A.5 to each other and to other *Sphingobacterium* species raises the possibility that our strains belong to different, yet unidentified, *Sphingobacterium* species. This assumption is also supported by the fact that the comparison of the genomic copies of the 16S rRNA genes of 1.A.4 and 1.A.5 shows only 97% similarity.

The sequences of our isolates potentially representing new species will contribute to a better understanding of the organization of *Sphingobacterium* genomes.

### Accession number(s).

The draft genome sequences of *Sphingobacterium* sp. 1.A.4 and 1.A.5 have been deposited in NCBI GenBank under accession numbers PEAZ00000000 and PEBA00000000, respectively.
